# Absolute and Relative Morbidity Burdens Attributable to Various Illnesses and Injuries Among Active Component Members of the U.S. Coast Guard, 2023

**Published:** 2024-06-20

**Authors:** Armed Forces Health Surveillance Division Defense Health Agency, US

## Abstract

**What are the new findings?:**

In 2023, injuries, mental health disorders, and musculoskeletal diseases constituted the categories of medical conditions associated with the most medical encounters, greatest numbers of members affected, as well as largest numbers of hospital days among active duty Coast Guard members, similar to DOD active component service members. Medical encounters increased by 13.3% when compared to last year, and major category conditions increased by 12.7% overall. In 2023 COVID-19 accounted for 0.4% of total medical encounters, a decrease from 1.4% in 2022, with no hospital bed days reported in 2023.

**What is the impact on readiness and force health protection?:**

The major condition categories in this report present health challenges for members of the U.S. Coast Guard and affect their service readiness. Loss of duty availability related to illness and injury reduces Coast Guard personnel readiness. Coast Guard members have unique occupational exposures that may benefit from specific risk reduction programs to mitigate these threats.

## 1 BACKGROUND

The U.S. Coast Guard (USCG) is the second smallest service of the U.S. Armed Forces, comprised of approximately 40,000 active component service members, and the only military service operating outside the authority of the Department of Defense (DOD). Between 2016 and 2021, USCG health care data were excluded from *MSMR*’s annual morbidity burden reports due to missing data.^1,2^ USCG personnel are eligible to use DOD health care facilities, but as many service members are not stationed near a DOD installation, the USCG operates primary care clinics in areas with sufficiently large Coast Guard populations. A higher proportion of civilian hospitalizations among USCG members has been noted,^1^ and this difference may extend to ambulatory care as well.

To quantify the impacts of various illnesses and injuries among members of the active component of the USCG in 2023, this summary employs the same disease classification system and morbidity burden measures used in the general active component burden analysis.

## 2 METHODS

The population for this analysis included all individuals who served in the active component of the USCG at any time during the surveillance period of January 1, 2023 through December 31, 2023. The methodology for summarizing absolute and relative USCG morbidity burdens in 2023 is identical to the methodology described on page 3 of this issue that was used to determine the absolute and relative burdens attributed to various illnesses and injuries among the active component of the U.S. Armed Forces.

## 3 RESULTS

In 2023 a total of 37,438 USCG service members had 442,798 total medical encounters, which included 8,917 bed days reported, for a rate of 0.24 bed days per USCG member who experienced at least 1 medical encounter (either ambulatory or hospitalization).

**Morbidity Burden, by Category**

In 2023, more active component USCG members (n=16,210 individuals affected) experienced medical encounters for injury than any other morbidity-related category (**Figure 1a**). Ranking third in terms of hospital bed days, this morbidity category accounted for over one-fifth (22.4%) of all medical encounters (**Figure 1b**).

Mental health disorders accounted for more hospital bed days (n=4,903) than any other morbidity-related category, comprising over half (55.0%) of all hospital bed days and ranking fifth in terms of numbers of individuals affected (**Figures 1a** and **1b**). Injury and mental health disorders combined accounted for over three-fifths (66.9%) of all hospital bed days and almost two-fifths (40.8%) of all medical encounters.

Maternal conditions, e.g., pregnancy complications and delivery, accounted for a relatively large proportion of all hospital bed days (n=1,202; 13.5%) but a much smaller proportion of total medical encounters (n=3,884; 0.9%) (**Figures 1a** and **1b**). Maternal conditions were the most prevalent medical condition among female active component USCG members. Women comprised approximately one-sixth (16.0%) of the active duty USCG in 2023.

**Medical Encounters, by Condition**

**Figure 2:**
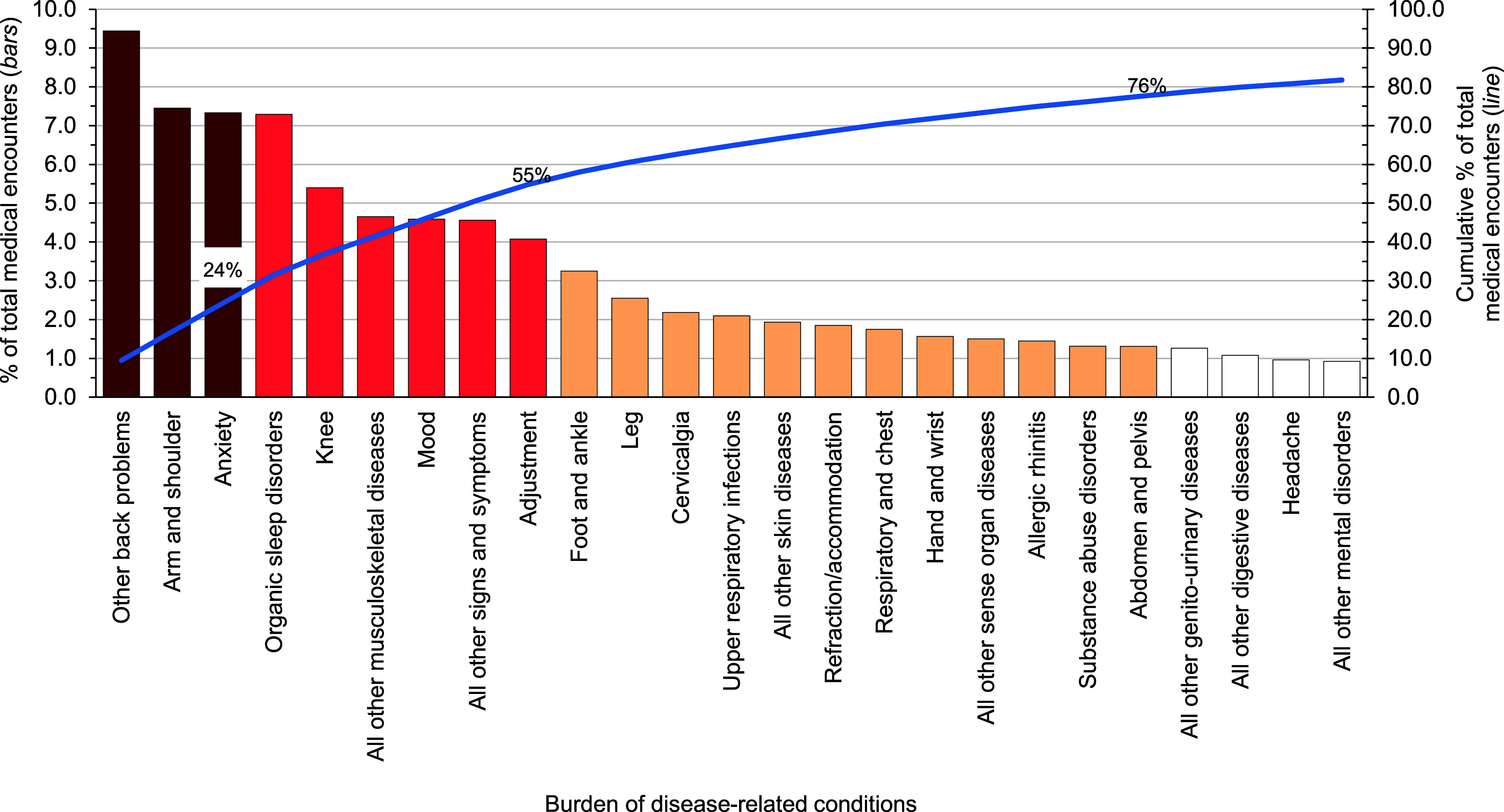
Percentage and Cumulative Percentage Distribution, Burden of Disease-related Conditions that Accounted for the Most Medical Encounters, Active Component, U.S. Coast Guard, 2023

In 2023, 5 disease-related conditions accounted for more than one-third (36.9%) of all illness- and injury-related medical encounters among active component USCG members: other back problems (includes lower back pain and other dorsalgia), arm/shoulder injuries, anxiety disorders, organic sleep disorders (e.g., obstructive sleep apnea, insomnia), and knee injuries (**Figure 2**). Moreover, the 10 conditions associated with the most medical encounters constituted more than half (58.0%) of all illness- and injury-related medical encounters.

The conditions in 2023 that accounted predominantly for medical encounters among active component USCG members were injuries, mental health disorders, and musculoskeletal diseases. Arm/shoulder (7.5%), knee (5.4%), foot/ankle (3.3%), and leg (2.6%) injuries contributed the most medical encounters (**Figure 2** and **Table**). Anxiety (7.3%), mood (4.6%), adjustment (4.1%), and alcohol/substance abuse disorders (1.3%) were the 4 most frequent mental health disorder diagnoses. Other back problems (9.4%), all other musculoskeletal diseases (4.7%), and cervicalgia (2.2%) constituted the most medical encounters among musculoskeletal disorders. COVID-19 accounted for 0.4% of total medical encounters during 2023.

**Individuals Affected, by Condition**

The 10 categories of conditions that affected the most USCG members in 2023 were all other signs and symptoms, refraction/accommodation, other back problems, upper respiratory infections, all other musculoskeletal diseases, organic sleep disorders, anxiety, all other skin diseases, arm and shoulder conditions, and respiratory and chest issues. COVID-19 affected 1,380 USCG members and ranked thirty-third for the number of individuals affected, a considerable decrease in rank from seventh in 2022.

**Hospital Bed Days, by Condition**

**Figure 3:**
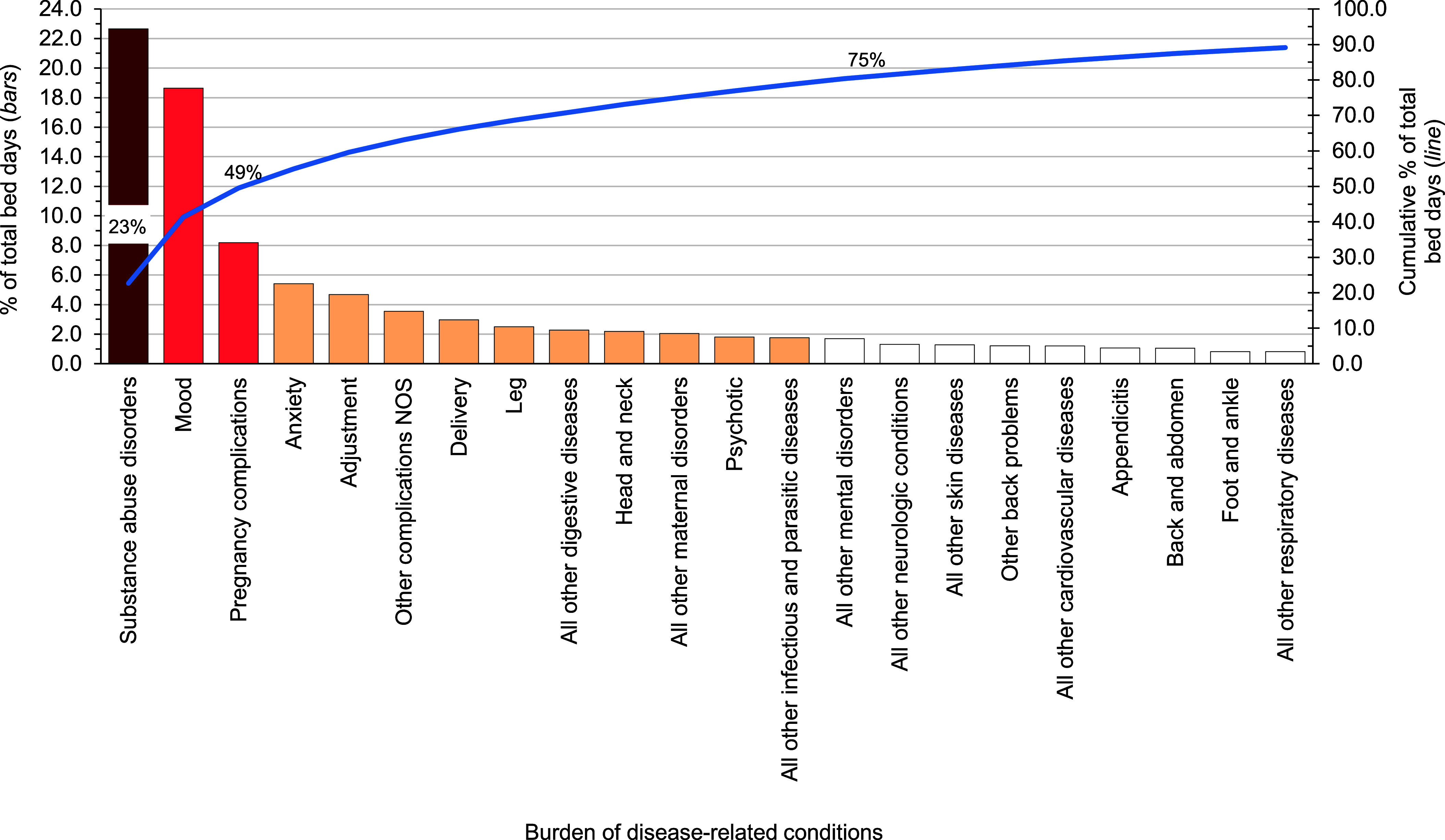
Percentage and Cumulative Percentage Distribution, Burden of Disease-related Conditions that Accounted for the Most Hospital Bed Days, Active Component, U.S. Coast Guard, 2023

In 2023, substance abuse and mood disorders accounted for about two-fifths (41.3%) of all hospital bed days (**Figure 3**). Four mental health disorders (substance abuse, mood, anxiety, adjustment) and 2 maternal conditions (pregnancy complications and delivery) combined accounted for more than three-fifths (62.5%) of all hospital bed days (**Table** and **Figure 3**). About 12% of all hospital bed days were attributable to injuries and poisonings. There were no hospitalizations due to COVID-19 among active component USCG members in 2023 (**Table**).

## 4 DISCUSSION

Health care utilization within the USCG was similar to the DOD when measured by total encounters and persons affected in 2023. The USCG rate was 11.8 encounters per person (442,798/37,438), compared to the DOD rate of 11.6 encounters per person (14,013,185/1,204,509). The USCG had a lower rate of hospitalization, however, with only 0.24 bed days per individual; the DOD reported 0.32 bed days per individual (390,181/1,204,509).

Compared to 2022, USCG medical encounters increased by 13.3%, with major category conditions reported increasing by 12.7% overall, and individuals affected and hospital bed days (8.5% and 2.3%, respectively) both rising as well. Mental health disorders resulted in more hospital stays than any other morbidity-related category, and mental health-related medical encounters increased by 21.5% compared to the previous year.

These reported increases may be overestimates, as 2022 was the first year DMSS data were archived and analyzed in the Military Health System (MHS) Information Platform (MIP), and during last year’s morbidity burden analysis completeness issues were identified for MHS GENESIS data being transferred to DMSS. Opportunities to address and resolve missing data issues that result from USCG hospitalizations in civilian facilities should be prioritized to accurately depict the true burden of disease in this population.

This report is consistent with the major findings of prior annual reports on morbidity burdens among active component U.S. service members. Injuries, mental health disorders, and musculoskeletal diseases were the categories of medical conditions associated with the most medical encounters, the largest numbers of affected service members, and the greatest numbers of hospital bed days. When examining ICD codes to the fourth digit character, USCG and DOD service members shared many disease-related conditions: other back problems within the musculoskeletal disease major diagnostic category; arm/shoulder and knee injuries within the injury major diagnostic category; anxiety disorders in the mental health disorder major diagnostic category; and organic sleep disorders within the neurologic condition major diagnostic category.

COVID-19 did not account significantly for medical encounters and bed days in 2023 compared to 2022. Besides the waning of the pandemic, active component service members represent a relatively young and healthy population less likely to experience severe consequences of COVID-19 infection.

Preventable illnesses and injuries, which contribute disproportionately to morbidity and health care burdens, should be high priority targets for intervention, research, and resources. In a 2018 survey, USCG members reported several mental health issues including serious psychological distress, failure to receive mental health services despite need, and other preventable risky health behaviors.^3^ Providing a matrix of major diseases each year enables the identification, in comparison with previous reports, of potentially avoidable health conditions among military personnel, and their proximate causes. Morbidity burden report findings can aid prioritization of effective interventions, provision of necessary care, and evaluation of their impacts and cost-effectiveness.^4^

## Figures and Tables

**Figure 1a:**
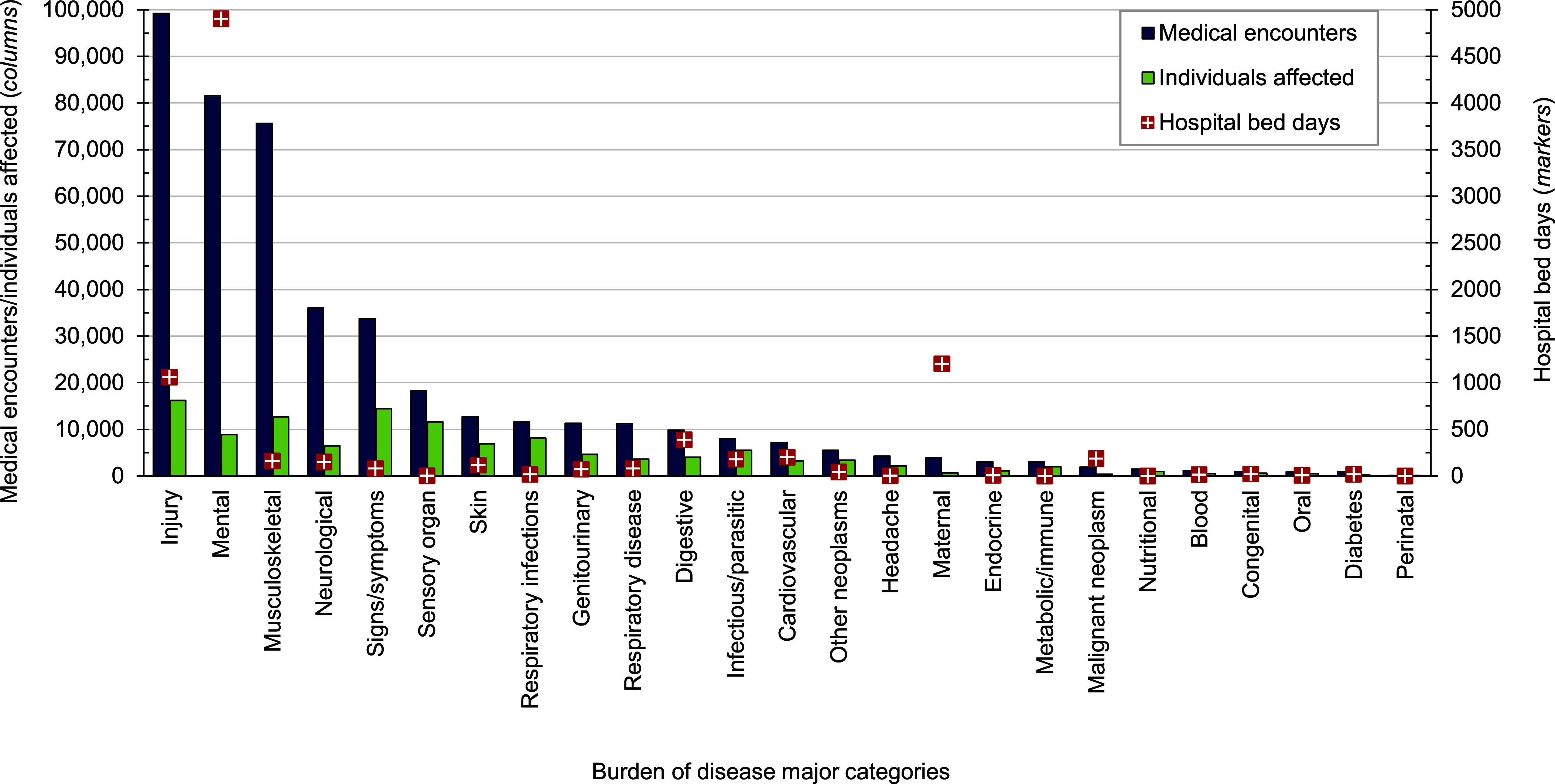
Numbers of Medical Encounters, Individuals Affected, and Hospital Bed Days by Burden of Disease Major Category, Active Component, U.S. Coast Guard, 2023

**Figure 1b:**
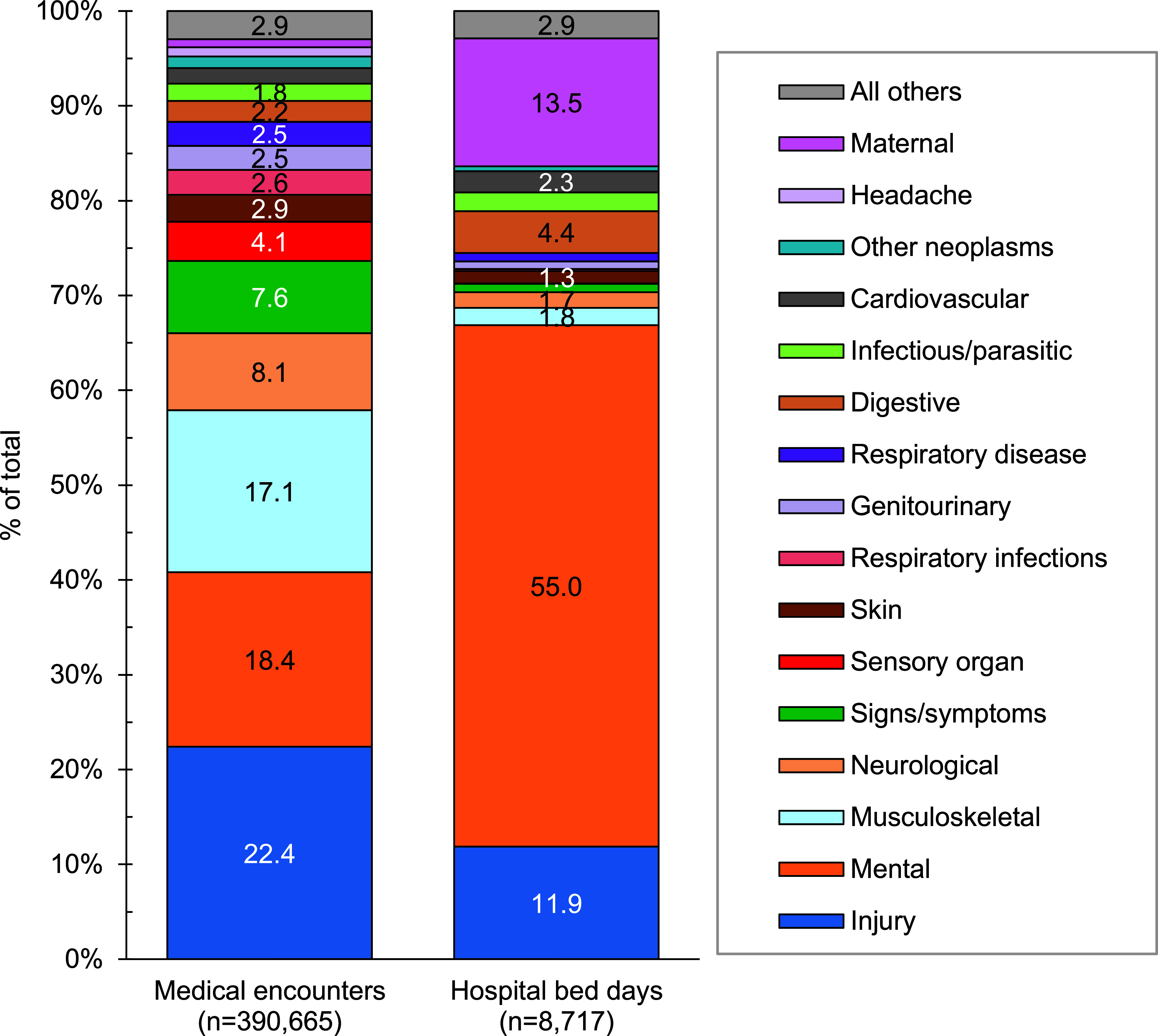
Percentage of Medical Encounters and Hospital Bed Days Attributable to Burden of Disease Major Categories, Active Component, U.S. Coast Guard, 2023

**Table:** Health Care Burdens Attributable to Various Diseases and Injuries, Active Component, U.S. Coast Guard, 2023

Major Category Condition^a^	Medical Encounters^b^		Individuals Affected^c^		Hospital Bed Days	
	No.	Rank^d^	No.	Rank^d^	No.	Rank^d^
**Injury and poisoning**	**99,167**		**23,348**		**1,060**	
Arm and shoulder	32,993	2	4,926	9	38	29
Knee	23,908	5	3,833	13	39	27
Foot and ankle	14,394	10	3,891	12	73	21
Leg	11,293	11	2,391	21	223	8
Hand and wrist	6,931	17	2,668	17	18	45
Head and neck	2,607	31	1,473	31	195	10
Back and abdomen	2,567	32	1,092	37	94	20
Unspecified injury	1,232	45	803	44	0	88
Environmental	997	52	798	45	3	73
Other complications NOS	927	55	498	56	316	6
Other harm from external causes	813	59	609	50	17	46
Poisoning, non-drug	244	93	184	85	6	63
All other injury	118	113	101	97	3	73
Other burns	68	119	43	109	0	88
Poisoning, drugs	62	123	31	115	35	31
Other superficial injury	13	135	7	134	0	88
**Mental health disorders**	**81,591**		**12,888**		**4,903**	
Anxiety	32,465	3	5,073	7	483	4
Mood	20,320	7	2,564	18	1,662	2
Adjustment	18,045	9	3,297	15	417	5
Substance abuse	5,810	20	548	54	2,020	1
All other mental disorders	4,090	25	1,090	38	151	14
Psychotic	301	88	37	112	161	12
Tobacco dependence	219	96	145	89	0	88
Personality	193	103	44	107	9	52
Somatoform	148	109	90	99	0	88
**Musculoskeletal diseases**	**75,597**		**17,036**		**161**	
Other back problems	41,803	1	7,111	3	108	17
All other musculoskeletal diseases	20,604	6	6,376	5	40	26
Cervicalgia	9,665	12	1,923	23	0	88
Osteoarthritis	1,931	38	986	39	6	63
Other knee disorders	727	66	269	75	7	56
Other shoulder disorders	695	68	302	70	0	88
Rheumatoid arthritis	172	105	69	105	0	88
**Signs, symptoms and ill-defined conditions**	**33,733**		**18,215**		**80**	
All other signs and symptoms	20,196	8	10,149	1	60	24
Respiratory and chest	7,733	16	4,740	10	11	50
Abdomen and pelvis	5,804	21	3,326	14	9	52
**Neurologic conditions**	**36,038**		**6,980**		**149**	
Organic sleep disorders	32,284	4	5,601	6	2	79
All other neurologic conditions	1,924	39	652	49	117	15
Chronic pain	940	54	398	62	27	34
Other mononeuritis, upper and lower limbs	510	72	227	81	0	88
Epilepsy	216	97	80	102	3	73
Multiple sclerosis	127	112	18	123	0	88
Parkinson's disease	37	128	4	136	0	88
**Sense organ diseases**	**18,246**		**13,552**		**4**	
Refraction/accommodation	8,178	15	7,133	2	0	88
All other sense organ diseases	6,651	18	4,248	11	4	69
Hearing disorders	2,637	30	1,676	26	0	88
Glaucoma	677	69	434	59	0	88
Cataracts	103	115	61	106	0	88
**Infectious and parasitic diseases**	**6,051**		**8,018**		**178**	
All other infectious and parasitic diseases	3,766	26	2,461	19	157	13
COVID-19	1,578	43	1,380	33	0	88
Tinea skin infections	1,206	48	920	40	0	88
Unspecified viral infection	611	71	569	53	0	88
STDs	380	83	303	69	0	88
Diarrheal diseases	352	85	311	67	21	39
Chlamydia	85	116	78	103	0	88
Intestinal nematode infection	14	134	11	129	0	88
Hepatitis B and C	11	137	7	134	0	88
Tropical cluster	7	140	4	136	0	88
Tuberculosis	5	142	4	136	0	88
Malaria	2	144	2	143	0	88
Bacterial meningitis	1	146	1	146	0	88
**Skin diseases**	**12,666**		**7,773**		**115**	
All other skin diseases	8,563	14	5,067	8	114	16
Sebaceous gland diseases	2,450	33	1,459	32	1	83
Contact dermatitis	1,653	42	1,247	34	0	88
**Respiratory diseases**	**11,221**		**4,421**		**80**	
Allergic rhinitis	6,415	19	1,484	30	0	88
All other respiratory diseases	1,833	40	1,128	36	73	21
Chronic sinusitis	1,207	47	740	46	3	73
Deviated nasal septum	715	67	411	61	0	88
Asthma	626	70	309	68	4	69
Chronic obstructive pulmonary disease	425	79	349	63	0	88
**Genitourinary diseases**	**11,279**		**5,807**		**73**	
All other genito-urinary diseases	5,583	22	2,730	16	19	41
Female genital pain	1,355	44	599	51	0	88
Menstrual disorders	1,211	46	695	47	7	56
UTI and cystitis	810	60	597	52	6	63
Kidney stones	764	61	279	74	28	33
Other breast disorders	752	63	431	60	1	83
Nephritis and nephrosis	306	87	121	91	12	49
Vaginitis and vulvitis	295	89	234	79	0	88
Benign prostatic hypertrophy	203	102	121	91	0	88
**Digestive diseases**	**9,797**		**5,042**		**390**	
All other digestive diseases	4,772	23	2,404	20	203	9
Esophagus disease	2,115	36	1,228	35	7	56
Other gastroenteritis and colitis	1,733	41	837	43	59	25
Constipation	430	78	295	72	5	67
Inguinal hernia	414	81	134	90	3	73
Appendicitis	225	95	97	98	95	19
Peptic ulcer disease	60	124	39	110	7	56
Cirrhosis of the liver	48	125	8	133	11	50
**Respiratory infections**	**11,567**		**8,671**		**18**	
Upper respiratory	9,278	13	6,939	4	4	69
Otitis media	1,191	49	872	41	1	83
Lower respiratory	1,098	51	860	42	13	48
**Cardiovascular diseases**	**7,206**		**3,554**		**201**	
All other cardiovascular diseases	3,496	27	1,665	27	107	18
Essential hypertension	2,915	28	1,567	28	1	83
Ischemic heart disease	439	75	158	86	61	23
Cerebrovascular disease	245	92	102	95	25	36
Inflammatory	75	117	31	115	7	56
Rheumatic heart disease	36	129	31	115	0	88
**Other neoplasms**	**5,493**		**3,952**		**44**	
All other neoplasms	2,748	29	1,894	24	27	34
Benign skin neoplasm	2,163	34	1,724	25	0	88
Lipoma	406	82	251	77	0	88
Uterine leiomyoma	176	104	83	101	17	46
**Headache**	**4,255**		**2,142**		**2**	
Headache	4,255	24	2,142	22	2	79
**Maternal conditions**	**3,884**		**1,237**		**1,202**	
Pregnancy complications	1,956	37	509	55	730	3
All other maternal disorders	1,104	50	314	66	182	11
Delivery	475	74	256	76	265	7
Ectopic/miscarriage/abortion	216	97	85	100	3	73
Puerperium complications	133	111	73	104	22	38
**Metabolic and immunity disorders**	**2,970**		**2,033**		**0**	
Lipoid metabolism disorders	2,153	35	1,563	29	0	88
Other metabolic disorders	422	80	239	78	0	88
Gout	320	86	187	84	0	88
Immunity disorders	75	118	44	107	0	88
**Endocrine disorders**	**3,027**		**1,249**		**8**	
Testicular hypofunction	909	56	297	71	0	88
Hypothyroidism	739	64	332	65	0	88
Other thyroid disorders	732	65	289	73	2	79
All other endocrine disorders	432	77	221	82	6	63
Polycystic ovarian syndrome	215	99	110	94	0	88
**Malignant neoplasms**	**1,875**		**371**		**185**	
Melanoma and other skin cancers	360	84	154	87	29	32
Colon and rectal cancers	264	90	19	122	20	40
Lymphoma and multiple myeloma	232	94	28	118	39	27
Brain	204	101	11	129	19	41
Breast cancer	159	106	26	119	2	79
Testicular cancer	155	107	34	113	5	67
All other malignant neoplasms	153	108	32	114	9	52
Leukemia	113	114	18	123	36	30
Liver cancer	68	119	2	143	25	36
Prostate cancer	65	122	9	132	0	88
Thyroid	43	127	12	127	1	83
Cervix uteri cancer	24	132	14	126	0	88
Trachea, bronchus, and lung cancers	19	133	4	136	0	88
Bladder cancer	8	139	4	136	0	88
Mouth and oropharynx cancers	7	140	3	141	0	88
Corpus uteri cancer	1	146	1	146	0	88
**Nutritional disorders**	**1,419**		**996**		**0**	
Overweight, obesity	982	53	657	48	0	88
All other nutritional disorders	436	76	338	64	0	88
Protein, energy malnutrition	1	146	1	146	0	88
**Blood disorders**	**1,128**		**593**		**15**	
All other blood disorders	490	73	217	83	8	55
Iron deficiency anemia	259	91	111	93	0	88
Other non-deficiency anemias	214	100	146	88	7	56
Hereditary anemias	140	110	102	95	0	88
Other deficiency anemias	25	131	17	125	0	88
**Diabetes mellitus**	**853**		**231**		**19**	
Diabetes mellitus	853	57	231	80	19	41
**Oral conditions**	**858**		**515**		**7**	
All other oral conditions	835	58	492	57	7	56
Dental caries	12	136	12	127	0	88
Periodontal disease	11	137	11	129	0	88
**Congenital anomalies**	**867**		**547**		**23**	
All other congenital anomalies	753	62	485	58	19	41
Congenital heart disease	67	121	38	111	0	88
Other circulatory anomalies	47	126	24	120	4	69
**Conditions arising during the perinatal period^e^**	**43**		**25**		**0**	
All other perinatal anomalies	36	129	20	121	0	88
Low birth weight	5	142	3	141	0	88
Birth asphyxia and birth trauma	2	144	2	143	0	88

Abbreviations: No., number; NOS, not otherwise specified; UTI, urinary tract infection; STDs, sexually transmitted diseases.

^a^ Burden of disease major categories and burden of disease-related conditions based on a modified version of those defined in the Global Burden of Disease Study.^5,6^

^b^ Medical encounters include total hospitalizations and ambulatory visits for the condition (with no more than 1 encounter per individual per day per condition).

^c^ Individuals with at least 1 hospitalization or ambulatory visit for the condition.

^d^ Rank is based on the number of encounters, individuals affected, or hospital bed days in respective columns within the listing of the 153 burden-related disease conditions. For individuals affected, 3 pairs of tied values (n=81; n=51; n=35) were given the same ranking (140; 145; 149). For hospital bed days, there were 9 conditions with the rank of 146 (0); 14 other conditions had tied rankings.

^e^ Conditions affecting newborns erroneously coded on service member medical records.

## References

[1] Pillai S, Chau M, Kamara I, Thomas D, Iskander J (2023). Hospitalizations among active duty members of the U.S. Coast Guard, fiscal year 2021.. MSMR..

[2] Armed Forces Health Surveillance Branch. (2016). Hospitalizations among members of the active component, U.S. Armed Forces, 2015.. MSMR..

[3] Pulkkinen AJ, My CG [Coast Guard] Let’s talk about your behavioral health..

[4] Devleesschauwer B, Maertens de Noordhout C, Smit GS (2014). Quantifying burden of disease to support public health policy in Belgium: opportunities and constraints.. BMC Public Health..

[5] World Health Organization. (2008). The Global Burden of Disease: 2004 Update..

[6] Murray CJL, Lopez AD (1996). The Global Burden of Disease: A Comprehensive Assessment of Mortality and Disability from Diseases, Injuries, and Risk Factors in 1990 and Projected to 2020..

